# Microneedling in Dermatology: A Comprehensive Review of Applications, Techniques, and Outcomes

**DOI:** 10.7759/cureus.70033

**Published:** 2024-09-23

**Authors:** Sharwari Jaiswal, Sugat Jawade

**Affiliations:** 1 Dermatology, Venereology and Leprosy, Jawaharlal Nehru Medical College, Datta Meghe Institute of Higher Education and Research, Wardha, IND; 2 Dermatology, Venereology and Leprosy, Datta Meghe Medical College, Datta Meghe Institute of Higher Education and Research, Nagpur, IND

**Keywords:** acne scars, collagen induction therapy, dermatology, microneedling, radiofrequency microneedling, skin rejuvenation

## Abstract

Microneedling, also known as collagen induction therapy, is a minimally invasive dermatological procedure that has gained widespread popularity for treating various skin conditions, including acne scars, wrinkles, hyperpigmentation, and stretch marks. By creating controlled micro-injuries in the skin, microneedling stimulates the body’s natural healing processes, resulting in increased collagen and elastin production, essential for maintaining skin elasticity and firmness. Over the past few decades, microneedling has evolved significantly, with advancements such as automated devices, radiofrequency microneedling, and combination therapies enhancing its effectiveness and safety profile. This comprehensive review explores the mechanisms of action, various techniques, and clinical applications of microneedling, highlighting its advantages over other skin rejuvenation methods. The review also examines patient satisfaction, safety considerations, and potential complications, providing a balanced perspective on its clinical utility. Furthermore, the discussion includes future directions in microneedling technology and research, focusing on emerging innovations and potential new applications. As the field advances, microneedling is poised to play an increasingly important role in aesthetic medicine, offering a reliable and effective solution for skin rejuvenation and beyond. This review is a valuable resource for healthcare professionals, guiding the optimization of microneedling practices and informing future research efforts.

## Introduction and background

Microneedling, also known as collagen induction therapy, is a minimally invasive dermatological procedure that involves using fine needles to create controlled micro-injuries in the skin [1]. These micro-injuries trigger the body’s natural wound-healing processes, leading to increased production of collagen and elastin, which are crucial for maintaining healthy, youthful skin [2]. The procedure is widely utilized to address various skin concerns, including acne scars, wrinkles, hyperpigmentation, and stretch marks. One of the key advantages of microneedling is its ability to stimulate skin rejuvenation while preserving the surrounding healthy tissue, resulting in quicker recovery and minimal downtime compared to other skin treatment techniques [3]. The versatility of microneedling allows it to be performed on almost any part of the body, though it is most commonly applied to the face, neck, and décolletage. Its growing popularity is attributed to its effectiveness, safety, and adaptability in treating dermatological issues [1].

The concept of microneedling can be traced back to ancient practices where sharp instruments were used for scar treatment and skin enhancement [1]. However, the modern form of microneedling began in the early 1990s when Dr. Andre Camirand, a plastic surgeon, noticed that scars improved after patients underwent tattooing without pigment. This observation led to the development of techniques that utilized needles to stimulate collagen production [4]. In 1997, Dr. Des Fernandes, a South African plastic surgeon, further advanced the field by creating the dermal roller, a cylindrical device embedded with fine needles, which became the forerunner of contemporary microneedling devices [5]. The introduction of the dermal roller provided more consistent results and significantly contributed to the adoption of microneedling in cosmetic dermatology [1]. Over the past two decades, microneedling has evolved significantly, with technological advancements leading to the development of automated devices, radiofrequency microneedling, and combination therapies that have further enhanced its efficacy. Today, microneedling is a well-established and widely used treatment in aesthetic medicine, supported by a growing body of scientific research that validates its safety and effectiveness [1].

This review aims to provide a comprehensive and current analysis of microneedling in dermatology, encompassing its mechanisms of action, applications, techniques, outcomes, and future directions. Despite its widespread use, there is a need for an in-depth synthesis of existing knowledge on microneedling to guide practitioners and inform clinical decision-making. The review seeks to fill this gap by critically examining the available evidence and offering insights into the nuances of microneedling as a therapeutic modality. As microneedling technology continues to advance rapidly and its range of applications expands, it is essential to understand the fundamental principles and clinical outcomes associated with the procedure. This review will explore the biological mechanisms that underpin microneedling’s effectiveness, the various devices and techniques employed, and the dermatological conditions for which microneedling is most beneficial. Additionally, the review will address patient satisfaction, safety considerations, and potential complications, providing a balanced perspective on the advantages and limitations of microneedling.

## Review

Mechanism of action

Microneedling is a minimally invasive dermatological procedure that utilizes fine needles to create controlled micro-injuries in the skin. This technique has gained significant attention for its effectiveness in treating various skin conditions, including scars, wrinkles, and overall skin rejuvenation. Understanding the mechanism of action of microneedling involves a detailed examination of skin anatomy, the biological responses it triggers, and how it compares to other skin rejuvenation techniques [1]. The skin comprises three primary layers: the epidermis, dermis, and subcutaneous tissue. The epidermis acts as a protective barrier, while the dermis contains collagen and elastin fibers that provide structural support and elasticity. Microneedling primarily targets the dermis, creating micro-wounds that stimulate the body’s natural healing processes without causing significant damage to the epidermis. This targeted injury promotes collagen synthesis and skin remodeling, making microneedling an effective treatment for various dermatological conditions [6]. One of the most significant biological responses to microneedling is collagen induction. The micro-injuries initiate a wound-healing cascade, releasing growth factors and activating fibroblasts responsible for producing collagen and elastin. Research has demonstrated that microneedling enhances collagen deposition and reorganization, improving skin texture and elasticity over time [7]. In addition to collagen induction, microneedling promotes neo-vascularization or the formation of new blood vessels. This process enhances blood supply to the treated area, improving nutrient delivery and supporting the healing process. Increased vascularity contributes to healthier, more vibrant skin and improves the healing of scars and other skin lesions [8]. Another significant advantage of microneedling is its ability to enhance transdermal drug delivery. The needles' micro-channels allow for better penetration of topical agents, such as serums containing vitamins or growth factors. This enhanced absorption can significantly increase the efficacy of these treatments, making microneedling a valuable adjunct in various dermatological therapies [9]. When comparing microneedling to other skin rejuvenation techniques, it is essential to consider their unique mechanisms and outcomes. Laser therapy, for example, uses focused light energy to target specific skin layers, often resulting in more significant thermal damage than microneedling [10]. While laser treatments can deliver dramatic results, they may also carry a higher risk of side effects, particularly in individuals with darker skin tones due to potential pigmentation changes. In contrast, microneedling is less invasive and is often preferred for patients with darker skin types [10]. Chemical peels involve the application of acidic solutions to exfoliate the outer layers of the skin. Although effective for addressing superficial skin issues, chemical peels can irritate and require downtime for recovery. Microneedling, on the other hand, allows for deeper skin penetration with minimal downtime, making it suitable for a broader range of skin types and conditions. Dermabrasion, another technique, mechanically sands the skin’s surface to remove the outer layers. This method can lead to significant downtime and is associated with a higher risk of complications. In comparison, microneedling offers a gentler alternative, promoting healing while minimizing damage to the epidermis, thereby reducing recovery time and potential adverse effects [11].

Techniques and devices

Microneedling has undergone significant advancements, with various techniques and devices developed to enhance its effectiveness and patient comfort. The three main types of microneedling devices include manual rollers, automated pen devices, and radiofrequency microneedling [3]. Manual rollers are handheld devices equipped with a roller covered in fine needles. These rollers are designed to create controlled micro-injuries in the skin and are often used at home, making them a popular choice for individuals looking to improve their skin texture. However, while they can address mild skin concerns, their limited needle depth often makes them less effective than professional devices [12]. In contrast, automated pen devices use a motorized mechanism to create thousands of microchannels in the skin, reaching depths up to 2.5 mm. This method allows for more precise treatment, effectively targeting scar tissue and promoting skin remodeling more efficiently than manual rollers [13]. Lastly, radiofrequency microneedling combines traditional microneedling with radiofrequency energy, utilizing thicker needles to deliver heat deep into the skin. This technique stimulates collagen production through thermal injury, improving skin tightening and texture with minimal downtime [13]. Before undergoing microneedling, several pre-procedure considerations are essential. Patients should provide their dermatologist with a comprehensive medical history, including details about any medications and allergies, to ensure safety and efficacy [14]. Scheduling the treatment in advance is also important, as patients may experience mild reactions and visible redness lasting a few days post-treatment. The microneedling procedure typically begins with applying a topical anesthetic to minimize discomfort. The selected device is then used to create small, controlled pricks across the treatment area, usually taking around 30 minutes. After the procedure, a soothing serum or lotion is often applied to cool the skin and enhance recovery [14]. Post-procedure care is crucial for achieving optimal results. Patients are advised to use a gentle moisturizer and avoid harsh products or sun exposure for a few days following treatment. Proper sterilization is essential for those using at-home derma rollers to prevent infection and ensure safety [15]. Recent advancements in microneedling technology have further enhanced its effectiveness and versatility. One notable development is the introduction of devices with adjustable needle depths, allowing practitioners to tailor treatments to specific skin concerns and individual patient needs. This customization can improve outcomes, as different skin issues may require varying penetration depths [16]. Additionally, microneedling can now be combined with other modalities, such as the application of platelet-rich plasma (PRP) or specialized topical serums. This combination approach enhances overall results and promotes faster healing and more significant improvements in skin texture and tone [17]. An overview of microneedling techniques and devices is provided in Table 1.

**Table 1:** Overview of microneedling techniques and devices

Technique/Device	Description	Advantages	Disadvantages
Manual Rollers [18]	Handheld cylindrical rollers with fine needles that penetrate the skin.	Affordable, widely available.	It requires more skill and a risk of uneven pressure.
Automated Pen Devices [19]	Motorized devices with adjustable needle depth for more controlled penetration.	Precision, adjustable depth, consistent results.	Higher cost requires training.
Radiofrequency Microneedling [20]	Combines microneedling with radiofrequency energy to enhance collagen induction.	Deeper skin tightening, effective for scars.	Expensive, more complex procedure.
Adjustable Needle Depth [21]	Devices that allow customization of needle depth based on skin condition.	Tailored treatments and reduced downtime.	Higher cost, more sophisticated device.
Combined Modalities (e.g., PRP, Topicals) [22]	Microneedling combines platelet-rich plasma (PRP) or other topicals to enhance outcomes.	Enhanced effectiveness promotes faster healing.	Requires additional substances, higher cost.

Clinical applications

Microneedling has emerged as a versatile treatment modality in dermatology, effectively addressing a wide range of skin concerns by inducing collagen production and promoting skin rejuvenation [23]. The procedure involves creating controlled micro-injuries in the skin, which stimulate the body’s natural healing response, resulting in increased collagen and elastin production. This process is particularly effective for treating atrophic acne scars, improving skin texture, and significantly reducing scar visibility over time [24]. Comparative studies have demonstrated that microneedling is often more effective than traditional treatments, such as chemical peels and laser therapy, in reducing acne scars, with higher patient satisfaction and a lower risk of side effects, particularly hyperpigmentation in darker skin types [25]. In addition to its efficacy in scar treatment, microneedling has significantly reduced the appearance of fine lines and wrinkles. The procedure helps firm the skin and create a youthful appearance by stimulating collagen production. Clinical studies indicate that patients often observe noticeable improvements within weeks following treatment [26]. Beyond wrinkle reduction, microneedling enhances overall skin texture and tone by promoting the regeneration of the dermis, leading to smoother skin and a more even complexion. These benefits make microneedling popular for comprehensive skin rejuvenation [26]. Microneedling has also shown promise in treating hyperpigmentation and melasma by disrupting pigment deposits within the skin. The micro-wounds created during the procedure improve the absorption of topical agents, thereby enhancing the efficacy of treatments for pigment disorders. Combining microneedling with topical treatments, such as vitamin C or retinoids, has been found to yield significantly better outcomes. This synergistic approach facilitates deeper penetration of these agents, leading to more effective management of pigmentation issues [27]. Moreover, microneedling treats hair loss conditions, such as androgenetic alopecia. The procedure stimulates the scalp, promoting increased blood flow and the release of growth factors that encourage hair regrowth. When combined with growth factor serums or PRP, microneedling can significantly improve results, leading to greater hair density and thickness [28]. The procedure also effectively reduces the appearance of stretch marks by promoting collagen production and skin remodeling in the affected areas. Patients frequently report a noticeable reduction in the visibility of striae following treatment. Comparative studies have shown that microneedling offers a safe and effective alternative to other treatments for stretch marks, such as laser therapy, often delivering comparable or superior outcomes with fewer side effects [29]. Additionally, microneedling has been effectively used to improve the appearance of surgical scars by promoting collagen remodeling and reducing scar tissue density. It has also shown efficacy in treating burn scars, helping to improve skin texture and elasticity in the affected areas. Furthermore, there is emerging evidence that microneedling may play a role in treating hyperhidrosis by disrupting the sweat glands. However, further research is needed to confirm its effectiveness in this area [30]. An overview of the clinical applications of microneedling is provided in Table 2.

**Table 2:** Clinical applications of microneedling

Clinical Application	Mechanism of Action	Efficacy	Combination Treatments
Acne Scarring [31]	Stimulates collagen production and skin remodeling.	Proven to reduce acne scar depth and improve texture.	platelet-rich plasma (PRP), topical retinoids, vitamin C, and laser treatments.
Skin Rejuvenation and Anti-aging [32]	Promotes collagen induction for wrinkle reduction and skin tightening.	Effective for fine lines, wrinkles, and overall texture improvement.	Hyaluronic acid, peptides, antioxidants.
Hyperpigmentation and Melasma [33]	Enhances absorption of depigmenting agents and promotes skin renewal.	Shows moderate efficacy in reducing pigmentation.	Topical depigmenting agents (hydroquinone), vitamin C.
Hair Restoration [34]	Increases blood flow to hair follicles and stimulates growth factors.	Improves hair density and promotes regrowth.	Minoxidil, growth factors, PRP.
Stretch Marks (Striae) [35]	Triggers collagen and elastin synthesis to reduce stretch mark visibility.	Effective in reducing the appearance of stretch marks.	PRP, topical treatments (retinoids).
Surgical and Burn Scars [36]	Enhances scar remodeling by stimulating collagen formation.	Improves scar texture and appearance.	Silicone gels, scar creams, laser treatments.
Hyperhidrosis [37]	Affects sweat glands by disrupting their function with microneedling.	Emerging evidence of efficacy in reducing sweating.	Botulinum toxin (Botox).

Safety and side effects

Microneedling is generally considered a safe procedure, though awareness of potential side effects and complications is essential for both patients and practitioners. This understanding facilitates informed decision-making and effective management of any adverse effects that may arise [38]. Common side effects of microneedling are typically mild and resolve within a few days. Erythema, or skin redness, is the most frequently observed reaction. It usually appears immediately after the procedure and lasts from a few hours to a few days. This redness is a normal response to the micro-injuries created during the treatment [1]. Mild edema, or swelling, may also occur in the treated area but generally subsides within a few days. Applying cold compresses can help alleviate discomfort associated with swelling. Another common side effect is transient hyperpigmentation, particularly in individuals with darker skin types. This condition, characterized by darkening skin in the treated area, usually resolves within a few weeks. Proper sun protection is crucial to prevent exacerbation [39]. While rare, some complications can occur. Infections are a potential risk, especially if proper hygiene and aftercare protocols are not followed. Signs of infection may include increased redness, warmth, swelling, and pus formation. Keep the treated area clean and avoid touching it with unwashed hands [40]. Allergic reactions can also occur, particularly when topical anesthetics or serums are applied during the procedure. Symptoms may include rash, itching, or hives; conducting a patch test before treatment can help identify potential allergies. Persistent scarring, though uncommon, can occur, particularly if the procedure is performed incorrectly or if the patient has a history of keloid formation. Selecting a qualified practitioner and adhering to post-procedure care is vital to minimize this risk [41]. Certain conditions may contraindicate microneedling. Patients with active skin infections, such as herpes simplex or impetigo, should treat these conditions before the procedure. Individuals with blood disorders, particularly those with clotting issues or those on anticoagulant therapy, should avoid microneedling. The safety of the procedure during pregnancy has not been established, so it is generally advised to postpone treatment until after childbirth. Additionally, individuals with a history of keloid scarring may be at increased risk for developing scars from the procedure [42]. Effective management of adverse effects is crucial for ensuring patient satisfaction and safety. Cold compresses and over-the-counter anti-inflammatory medications, such as ibuprofen, can help reduce redness and swelling for erythema and edema. In cases of hyperpigmentation, broad-spectrum sunscreen and topical agents containing ingredients like hydroquinone or retinoids can help manage and prevent further pigmentation issues [43]. If signs of infection develop, prompt medical attention is necessary, and topical or oral antibiotics may be prescribed. For allergic reactions, it is important to discontinue the use of any suspected allergens and consult a healthcare provider for appropriate treatment. In cases of persistent scarring, a referral to a dermatologist for further evaluation and treatment options, such as laser therapy or corticosteroid injections, may be warranted [44].

Patient satisfaction and outcomes

Microneedling has gained recognition for its clinical effectiveness and the level of patient satisfaction it delivers. Understanding treatment outcomes and satisfaction factors is key to optimizing patient care and managing expectations [45]. Objective measurements are essential in evaluating the effectiveness of microneedling. Common assessment tools include photographic analysis, clinical scoring systems, and skin biopsies. Before-and-after photographs are frequently utilized to visually assess improvements in skin texture, tone, and the appearance of scars or wrinkles [46]. Standardized scales such as the Vancouver Scar Scale (VSS) or the Global Aesthetic Improvement Scale (GAIS) quantify changes in skin conditions. In some studies, skin biopsies measure collagen density and histological changes post-treatment [46]. Patient-reported outcomes (PROs) provide valuable insights into the subjective experience of the treatment. Measures such as quality-of-life assessments, satisfaction surveys, and self-assessment questionnaires are commonly used [47]. Tools like the Dermatology Life Quality Index (DLQI) assess the impact of skin conditions on daily life and overall well-being. Patients often rate their satisfaction with aspects of the treatment, including pain, recovery time, and aesthetic results. Additionally, self-assessment questionnaires may gauge perceptions of improvement in skin appearance and texture [47]. Several factors can significantly affect patient satisfaction with microneedling outcomes. Treatment response can vary widely, as different skin types react differently to microneedling. For example, individuals with oily or acne-prone skin may experience more pronounced improvements in scarring compared to those with dry or sensitive skin. Patients with darker skin types may be more susceptible to post-inflammatory hyperpigmentation, which can influence overall satisfaction with the treatment [48]. Managing realistic expectations is crucial for patient satisfaction. Educating patients about the procedure, expected results, and potential side effects is essential. Open communication between patients and providers regarding goals and anticipated outcomes can improve satisfaction. Providers should clarify that multiple sessions may be required to achieve optimal results [49]. Understanding the long-term efficacy of microneedling and the need for maintenance treatments is important for both patients and practitioners. Studies indicate that microneedling results can last several months to years, depending on factors such as skin type, the condition being treated, and the depth of the treatment. The collagen remodeling process continues for months post-treatment, leading to gradual improvements. However, aging and sun exposure can affect the durability of results [1]. To maintain results, many patients benefit from maintenance treatments every six to 12 months, with frequency varying based on individual skin conditions and goals. Microneedling is often combined with other treatments, such as PRP or specialized topical serums, to enhance outcomes and prolong effects [50].

Future directions and research

One of the most exciting advancements in microneedling is the development of nanoneedling. This cutting-edge technique employs ultra-fine, nano-sized needles that create micro-channels with minimal trauma, thereby enhancing the delivery of therapeutic agents [51]. Nanoneedling's ability to penetrate the skin with smaller, more controlled injuries may improve treatment outcomes for various skin conditions, including acne scars and hyperpigmentation. Its reduced invasiveness often results in shorter recovery times and less patient discomfort [51]. Another promising area is the integration of microneedling with regenerative medicine techniques. Combining microneedling with stem cell treatments or PRP can amplify the body’s natural healing processes. This synergistic approach holds significant potential for improving outcomes in conditions like androgenetic alopecia and chronic wounds. As regenerative medicine advances, microneedling’s role in facilitating tissue repair and rejuvenation could revolutionize dermatological treatment protocols [52]. Beyond current uses, microneedling shows promise in novel medical applications, particularly in gene therapy delivery. Recent research suggests microneedles can effectively serve as a transdermal delivery system for genetic material, enabling targeted treatment for skin disorders, cancers, and potentially systemic diseases. By creating microchannels in the skin, microneedling enhances the absorption of therapeutic agents, positioning it as a valuable tool in the evolving field of gene therapy [53]. Additionally, there is an exploration into microneedling's potential for treating systemic diseases. Traditionally used for dermatological issues, its capacity for transdermal drug delivery could open new possibilities for managing conditions such as diabetes, cardiovascular diseases, and neurological disorders. This expansion into systemic applications could significantly broaden the impact and utility of microneedling in clinical practice [54]. As microneedling technology and applications progress, clinical trials are essential to establish standardized protocols and guidelines. Well-designed studies will be critical in determining optimal treatment parameters, including needle depth, frequency, and device selection for various skin types and conditions. Moreover, further research is needed to assess the long-term safety and efficacy of microneedling, particularly concerning potential adverse effects and overall patient satisfaction [16]. Future directions and research in microneedling are summarized in Table 3.

**Table 3:** Future directions and research in microneedling

Future Direction	Description	Potential Benefits	Research Gaps
Nanoneedling [55]	Use ultra-fine needles (smaller than standard microneedling) to target superficial layers.	Minimally invasive, faster recovery, precise treatment.	Lack of long-term studies on efficacy and safety.
Integration with Regenerative Medicine [56]	Combining microneedling with stem cells and growth factors for enhanced skin regeneration.	Accelerated healing and enhanced tissue repair.	Limited clinical trials and standardization.
Gene Therapy Delivery [57]	Microneedling is a potential route for delivering gene therapy agents into the skin.	Targeted therapy for genetic disorders and skin diseases.	Early-stage research, ethical considerations, and safety.
Microneedling in Systemic Diseases [58]	Exploring the use of microneedling for transdermal drug delivery in systemic conditions.	Non-invasive drug delivery, a potential treatment for chronic diseases.	Limited data on long-term systemic effects and efficacy.
Combination with Advanced Modalities [59]	Exploring advanced combinations like microneedling with lasers, radiofrequency, and PRP.	Enhanced results, multi-faceted treatment approaches.	Need for more comparative studies and optimization.
Ongoing Clinical Trials [60]	Studies investigating new applications, safety profiles, and optimization of microneedling techniques.	Improved treatment protocols and expanded applications.	Need for more robust, large-scale clinical trials.

## Conclusions

In conclusion, microneedling has emerged as a versatile and effective dermatological procedure with many applications, from treating acne scars and wrinkles to enhancing skin texture and tone. Its minimally invasive nature and ability to stimulate the body’s natural healing processes have made it a popular choice among patients and practitioners alike. The procedure's evolution, driven by advancements in technology and technique, has further enhanced its efficacy and safety profile, solidifying its role in modern aesthetic medicine. As research continues to explore new applications and refine existing methods, microneedling's potential for skin rejuvenation and beyond will likely expand, offering even greater patient benefits. This comprehensive review underscores the importance of understanding the mechanisms, techniques, and clinical outcomes associated with microneedling to optimize its use and ensure the best possible results. Looking ahead, ongoing innovation and rigorous scientific investigation will be crucial in unlocking the full therapeutic potential of microneedling in dermatology.
